# Genetics of Dementia with Lewy Bodies in a Chinese Population

**DOI:** 10.1002/alz.087846

**Published:** 2025-01-03

**Authors:** Lu Shen, Xiaoli Hao, Xuewen Xiao, Qijie Yang, Bin Jiao

**Affiliations:** ^1^ Xiangya Hospital, Central South University, Changsha, Hunan China

## Abstract

**Background:**

Genetics plays an important role in dementia with Lewy bodies (DLB) and remains poorly understood. Previous research has identified several genes associated with DLB, including APOE, GBA, SNCA, BIN1, TMEM175, PLCG2, and CNTN1. To date, genetic studies on DLB have focused on Caucasian population. The gaps in DLB genetic study on East Asian populations need to be filled. Therefore, we conducted a comprehensive genome‐wide association study (GWAS) to identify genetic risk factors for DLB in China.

**Method:**

We performed whole‐genome sequencing on a cohort of 143 patients with DLB and 2007 healthy controls. Common variant‐based association analysis was conducted using PLINK 1.9, while gene‐based association tests for rare variants were performed using the sequence kernel association test‐optimal. Adjustments were made for gender, age, and principal components. Additionally, PLINK 1.9 was used to test variants associated with the onset age of DLB.

**Result:**

Our results confirmed previously reported associations with common variants to a certain extent, particularly the APOE gene (rs429358, p = 1.71 × 10^–10^). No rare‐damaging variants were significantly associated with DLB in our cohort. In the rare‐missense‐variant groups, the combined effect for USP13 gene showed suggestive association with DLB at p‐value < 1 × 10^−4^ (p = 7.75 × 10^‐5^). The protein encoded by the USP13 gene has been considered a major contributing factor to the presence of Lewy bodies based on previous research. No variants were associated with the onset age of DLB.

**Conclusion:**

Despite the small sample size for a genome‐wide association study, we present the first comprehensive study in dementia with Lewy bodies in China so far. These data show that common genetic variability has a role in the disease. Further replication study of USP13 gene will be important.

